# Invasive Group A streptococcal infections: are we facing a new outbreak? A case series with the experience of a single tertiary center

**DOI:** 10.1186/s13052-023-01494-9

**Published:** 2023-07-19

**Authors:** Nicolò Garancini, Giulia Ricci, Michele Ghezzi, Paola Tommasi, Fiammetta Zunica, Anna Mandelli, Elena Zoia, Enza D’Auria, Gian Vincenzo Zuccotti

**Affiliations:** 1 Pediatric Department, “Vittore Buzzi” Children’s Hospital, 20154 Milan, Italy; 2 Division of Pediatric Anesthesia and Intensive Care Unit, Department of Pediatrics, Children’s Hospital Vittore Buzzi, 20154 Milan, Italy; 3 Department of Biomedical and Clinical Science, Università degli Studi di Milano, 20157 Milan, Italy

**Keywords:** Streptococcus pyogenes, iGAS infection, Children, Epidemiology, Case series

## Abstract

**Background:**

In pediatric age, Group A Streptococcus (GAS) is responsible for a wide spectrum of clinical manifestations, from mild localized infections to life-threatening invasive diseases. In December 2022, the World Health Organization reported an increased incidence of scarlet fever and invasive GAS infections (iGAS) cases in Europe and the United States. In line with these observations, surveillance has been strengthened in our Region, allowing the identification of certified or highly suspected forms of iGAS.

**Case presentation:**

We report here 4 emblematic cases of iGAS admitted to our Intensive Care Unit (ICU) in the short time span from mid-February to mid-March 2023. Particularly, we describe a case of pleuropneumonia (4 year old boy) and a case of respiratory failure (2 year old boy), who necessitated Non-Invasive Ventilation support, a case of Streptococcal Toxic Shock Syndrome (6 year old girl), presenting with multi-organ failure, who needed Invasive Ventilation, and a case of meningitis (5 year old girl). All these patients needed intensive care support.

**Conclusions:**

Accurate differential diagnosis and early treatment both could help to reduce the transmission of GAS and consequently the risk of severe iGAS. These cases confirmed the need for close monitoring and appropriate notification, in order to verify their actual increased incidence.

**Supplementary Information:**

The online version contains supplementary material available at 10.1186/s13052-023-01494-9.

## Background

*Streptococcus pyogenes* (GAS, Group A Streptococcus) is a human-specific bacterial pathogen, responsible for a wide spectrum of clinical manifestations. The most common forms in the pediatric age group are mild localized infections, such as tonsillitis, pharyngitis, impetigo, cellulitis and scarlet fever. More rarely, it can cause life-threatening invasive diseases, burdened by important mortality and morbidity, such as necrotizing fasciitis and Streptococcal Toxic Shock Syndrome (STSS); in addition, ineffective treatment exposes to post-infectious sequelae, such as post-streptococcal glomerulonephritis or rheumatic disease [1]. Diagnosis of GAS infections can be challenging, considering that most of these clinical conditions overlap with non-GAS infections. Therefore, especially for pharyngitis, several diagnostic tools have been developed, like clinical scores (e.g., McIsaac scoring system or the modified Centor score), Rapid Antigen Detection Tests (RADT) and Nucleic Acid Amplification Techniques (NAAT). Nevertheless, throat culture is currently considered the reference standard for diagnosing GAS infections [2].

In December 2022, the World Health Organization (WHO) reported an increased incidence of scarlet fever and invasive Group A Streptococcus infections (iGAS) cases in Europe and the United States. Considering that neither an increased antibiotic resistance nor a new type of *emm* gene sequence (associated with M protein production and responsible for the more invasive forms [3]) have been reported, WHO currently assesses the risk of iGAS for the general population to be low [4].

In line with these findings, in our Region, surveillance has been strengthened with increased testing in children aged 3–12 years with suspicious symptoms of scarlet fever and iGAS, by the use of RADT, both in Emergency Department and Primary Care. Despite the end of most of anti-COVID-19 restrictions, the current number of notifications is still lower than in the pre-pandemic period; nonetheless, in line with what has been observed nationwide and in some European countries, there has been a significant increase in 2022, with a doubling of cases compared to 2021. This trend seems to be confirmed, considering that the notifications of scarlet fever cases almost reached the total number of the entire previous year in the month of January 2023 only [5].

This surveillance system allowed the identification of certified or highly suspected forms of iGAS. We report here some emblematic cases over the short time span from mid-February to mid-March 2023.

## Case presentation

### Case 1—pleuropneumonia

G., a 4 years-old child with no major clinical history, recently came in contact with a case of streptococcal pharyngitis. He was later clinically diagnosed with scarlet fever and treated with amoxicillin-clavulanic acid, with regression of symptoms. Five days later he came back to the Emergency Department for fever and left arm pain: blood tests were performed, showing neutrophilic leukocytosis and mild CRP elevation. He was discharged with anti-inflammatory therapy and orthopedic re-evaluation was indicated.

The following day, the patient was found uncomfortable, with difficulty in maintaining sitting position due to intense cervical and left upper limb pain, in mild respiratory distress with conserved normal oxygen saturation in room air. Blood analysis showed neutrophilic leukocytosis, high CRP levels, coagulation testing highlighted PT and aPPT elongation in known factor XI deficiency; several blood cultures were collected, later found to be negative. Chest radiograph showed mild thickening in the left retrocardiac side with partial loss of the ipsilateral hemidiaphragm border. Rapid Antigen Test for GAS was positive.

**Fig. 1:**
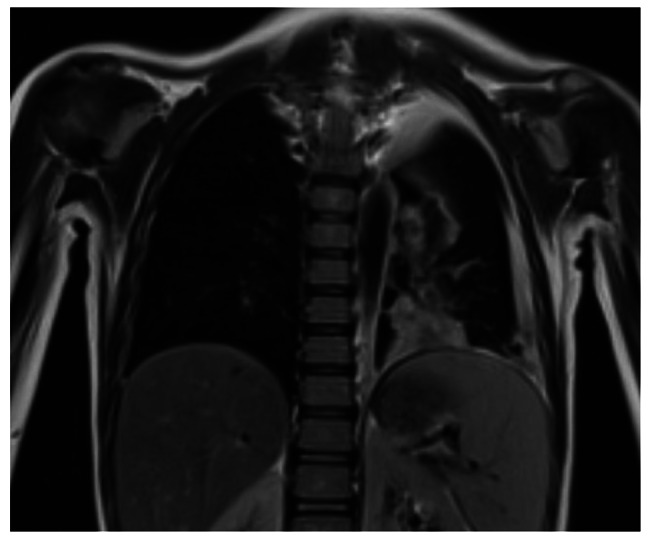
Pulmonary MRI, extensive pulmonary thickening in the left retrocardiac site, with homolateral pleural effusion

Due to the need to administer pre-procedure prothrombin complex concentrate, diagnostic lumbar puncture was postponed and, in suspicion of meningitis, intravenous ceftriaxone and acyclovir were started. Because of the progressive worsening of G. general condition, cerebral and pulmonary MRI was performed, showing no brain parenchymal damage nor abnormal contrast agent uptake, but finding an extensive pulmonary thickening in the left retrocardiac site, with homolateral pleural effusion [Fig. 1]. Due to clinical and radio-echographic worsening, clarithromycin was added and a chest drainage was performed, with discharge of abundant turbid pleural fluid, with negative culture tests. Non-invasive ventilation with CPAP and respiratory physiotherapy were also necessary.

Thereafter the patient clinically improved significantly, with resolution of the irradiated pleuritic pain and fever.

### Case 2—respiratory failure

M., a previously healthy 2-year-old boy, was admitted to an Emergency Department with fever and significant respiratory distress. Blood analysis showed marked leukocytosis, mild CRP increase; nasopharyngeal swab test for SARS-CoV-2, Influenza A/B and RSV was negative. Chest X-ray showed a small right supra-basilar thickening. M. was initially treated with oxygen, oral corticosteroid and respiratory support with High Flow Nasal Cannula. Because of the persistent respiratory distress, he was transferred to our Pediatric ICU, where he started non-invasive ventilation with CPAP, intravenous corticosteroid therapy and, after blood culture sampling, later found to be negative, parenteral antibiotic therapy with ampicillin-sulbactam. Rapid Antigen Test for GAS was positive.

M. general condition gradually improved, he was transferred to our Pediatric department and then a few days later discharged, after the complete resolution of his respiratory distress.

### Case 3—streptococcal toxic shock syndrome (STSS)

R., a 6-year-old girl with no previous clinical history, was admitted to an Emergency Department for cough, fever and progressive worsening of general condition. She presented polypnea, low oxygen levels (SpO2 < 90% in room air) and absent vesicular murmur in the left mid-basal field; scarlatiniform rash was present. Blood tests showed metabolic acidosis with hyperlactacidemia, leukopenia with moderate neutropenia (WBC 1030/mmc, Neu 640/mmc), high levels of CRP and procalcitonin and significant alteration of renal function. Chest X-ray showed a left mid-basal thickening with pleural effusion. After performing blood culture, ceftriaxone and supportive therapy was started but, due to progressive altered mental status, she was transferred to our Pediatric ICU.

**Fig. 2:**
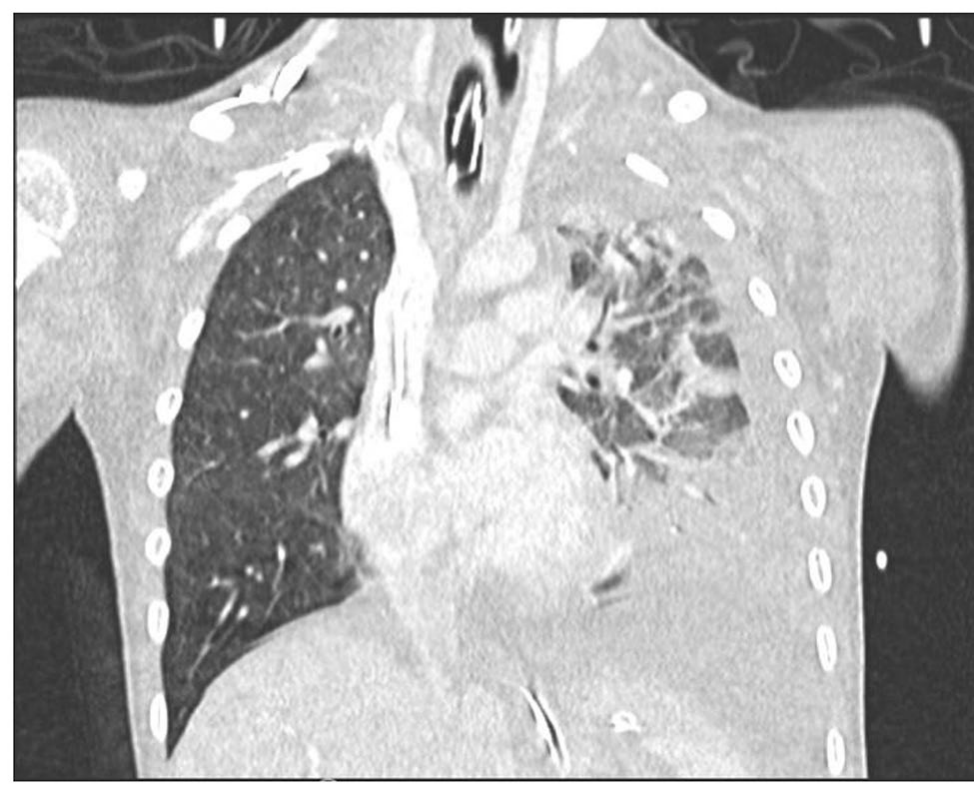
Chest CT scan, extensive consolidation of the left lower lobe

**Fig. 3:**
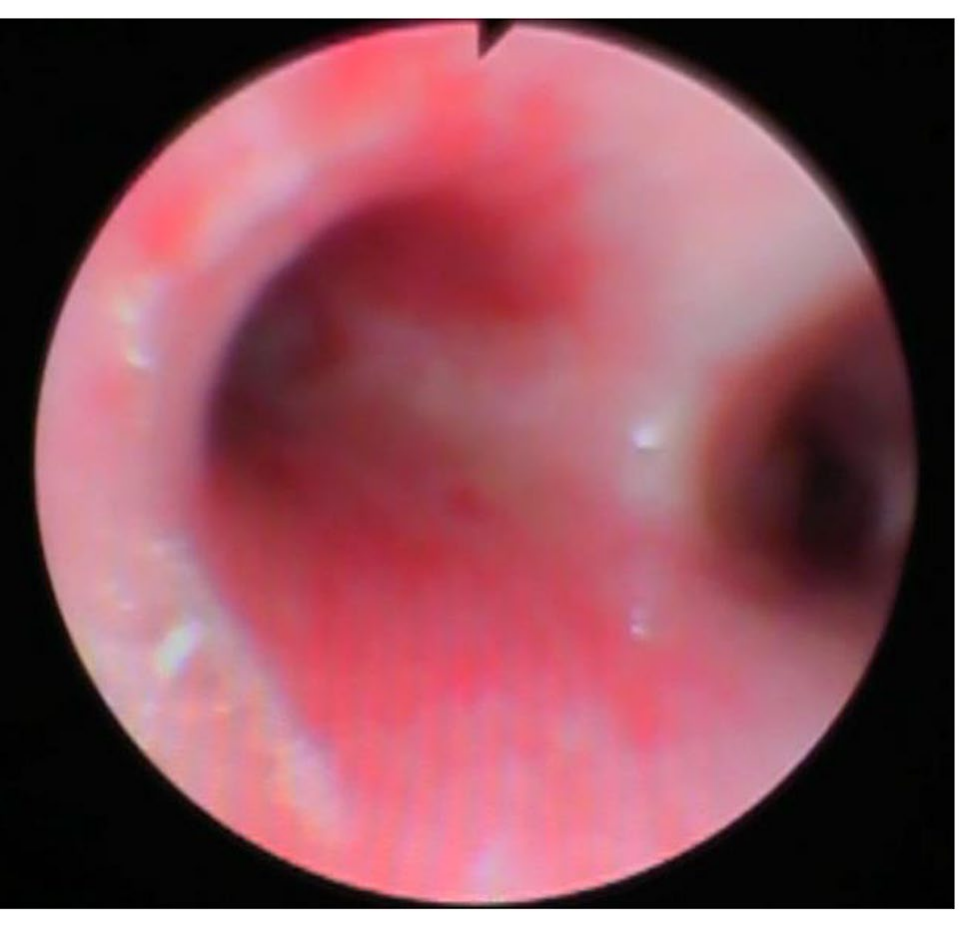
Bronchoscopy, necrotizing pneumonia with hemorrhagic alveolitis

On admission, after a brief stable phase with CPAP respiratory assistance, R. presented a new cardio-respiratory worsening, with myocardial dysfunction (diffuse cardiac hypokinesia, Left Ventricular Ejection Fraction 33%). Volemic loading, amine support, orotracheal intubation with subsequent mechanical ventilation and placement of chest drainage were necessary. On chest CT scan, extensive consolidation of the left lower lobe with air bronchogram and markedly hypovascularized areas, consistent with necrotizing pneumonia was found; apico-basal pleural effusion and apical atelectasis in the left upper lobe was also revealed [Fig. 2]. Bronchoscopyconfirmed the necrotizing pneumonia with hemorrhagic alveolitis [Fig. 3].

Rapid Antigen Test for GAS was positive. Given the suspicion of STSS, ev antibiotic therapy was empirically modified with clindamycin (for its anti-toxin effect) and vancomycin (given the clinical difficulty in distinguishing streptococcal from staphylococcal shock), the latter replaced with penicillin G upon confirmation of positive blood culture for Streptococcus pyogenes. Supportive treatment with Intravenous Immunoglobulinwas necessary [6] and, after discussion with pediatric infectiologist, high-dose steroid therapy was started.

During the following days of hospitalization, the patient presented a gradual improvement in general condition with normalization of blood count, inflammatory indices, renal and cardiac function; respiratory status also improved with possibility of endotracheal tube removal and, after a transient phase of Non Invasive Ventilation, resumption of spontaneous breathing in room air.

### Case 4—meningitis

**Fig. 4:**
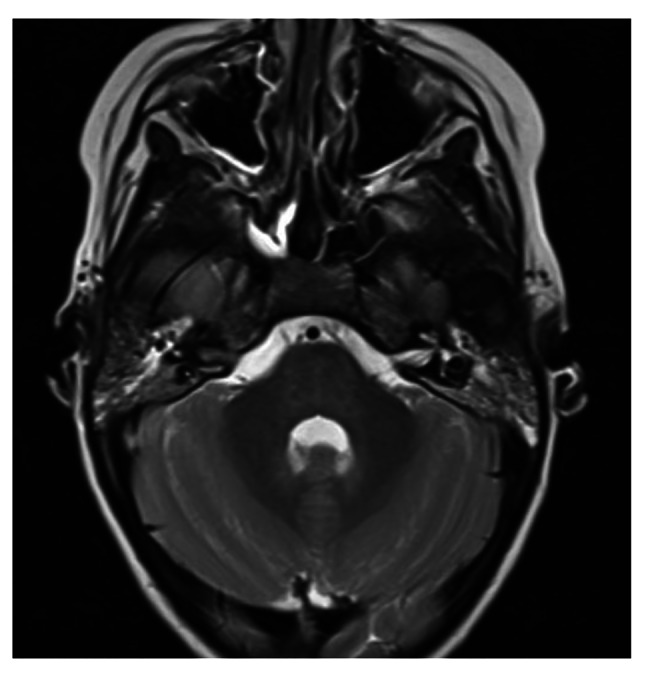
Brain MRI, diffusely salient cortical CSF spaces in supra- and subtentorial location, contrast enhancement of both petro-mastoid complexes

E., a 5-year-old girl with no history of major clinical events, presented to our Emergency Department for fever, irritability, headache and vomiting. On arrival, she was confused (Glasgow Coma Scale 14, Eye 4—Motor 6—Verbal 4), clinical examination showed signs of meningeal irritation, such as nuchal rigidity and positive Lasegue’s sign. Due to the presence of tonsillar hypertrophy with exudate, Rapid Antigen Test for GAS was performed, resulting positive. Blood tests showed significant neutrophilic leukocytosis, elevation of inflammatory indices and significant elongation of INR. Given the parental refusal to perform plasma transfusion due to religious reasons, diagnostic lumbar puncture was not performed. However, in suspicion of meningitis, empirical antibiotic therapy with ceftriaxone was started (after performing blood culture, later found to be negative). MRI showed diffusely salient cortical Cerebro-Spinal Fluid (CSF) spaces in supra- and subtentorial location, in the absence of pathologic endocranial contrast-enhancement; both petro-mastoid complexes presented T2-hyperintense material with contrast-enhancement. EEG showed a diffuse slow pattern both in sleep and wakefulness [Fig. 4].

During the subsequent hospitalization, after diagnosis and correction of a specific factor VII deficiency, it was possible to perform a lumbar puncture: CSF analysis showed significant hypoglycemia (4 mg/dl) and leukorrhea with predominance of polymorphonucleocytes; bacterioscopic and culture negative, undergoing antibiotic therapy.

During the subsequent hospitalization, E. presented gradual normalization of both neurological status and inflammatory indices; EEG and control MRI also improved.

## Discussion and conclusions

This case series describes different iGAS, which occurred from mid-February to mid-March 2023 at our Center. These cases came to our attention after WHO reported [3] and confirmed the need for close monitoring and appropriate notification, in order to verify their actual increased incidence. Since the end of 2022, reports of increased prevalence of iGAS in children have appeared in England [7]. Similar concerns are also being reported in the Netherlands, France, Ireland and the United States [8–12].

In agreement with epidemiological data the age range of our patients was 2–6 years [7].

Our findings are in line with the WHO report and confirm the need for close monitoring and appropriate notification, in order to verify their actual increased incidence. After 2 years of low incidence, the resurgence of predisposing viral infections may also have amplified this apparent new increase in iGAS cases. The most frequently reported viral infection associated with iGAS is Varicella Zoster Virus infection [13]. In our laboratory VZV is not routinely tested on nasopharyngeal swab: nonetheless, among our patients, those with the most critical clinical presentation presented a viral coinfection, particularly for Metapneumovirus (Case 1), Coronavirus and Enterovirus/Rhinovirus (Case 3); one patient was not tested (Case 4).

Of note, only one of our patients had the confirmation of iGAS with a positive blood culture. All the others were strongly suspected, based on clinical presentation and positive RADT, which has been demonstrated to have a good specificity [14]. This allowed to timely start antibiotic therapy, which is critical to effectively treat iGAS.

In conclusion, an accurate differential diagnosis combined with an early treatment both could help to reduce the transmission of GAS and consequently the risk of severe iGAS infection. Early recognition of iGAS disease and prompt initiation of specific and supportive therapy for patients can be lifesaving. Close contacts of iGAS cases should also be identified, assessed and managed according to national guidelines. As stated by WHO, Public Health authorities should consider activities to raise awareness among clinicians and the general public and encourage prompt testing and treatment of iGAS infections [15]. Lastly, even if GAS has remained highly susceptible to antimicrobial agents and WHO does not currently report an increased antibiotic resistance, decreased susceptibility to β-lactams due to PBP2x mutation has been recently demonstrated and should then be monitored in order to evaluate its potential clinical impact [16]. In addition, in case of allergy or therapy failure, macrolides or lincosamides are generally considered to be alternative options but resistance against them may also become a problem, since it has already emerged in numerous countries, like Greece [17], Hungary [18] and especially China [19].

## Electronic supplementary material

Below is the link to the electronic supplementary material.

Supplementary Material 1

## Data Availability

The datasets used and/or analyzed during the current study are available from the corresponding author on reasonable request.

## Declarations

No experimental protocols have been applied in the manuscript. The study was exempt from ethical committee approval since it was confinedto anonymized and unidentifiable data routinely collected in our clinicalpractice. All methods were performed in accordance with the ethical standards as laid down in the Declaration of Helsinki and its later amendments or comparable ethical standards. An informed consent was obtained from the parents of the patients to either describe the case report and use the pictures.

## List of abbreviations

aPTT: Activated Partial Thromboplastin Clotting Time
CPAP: Continuous Positive Airway Pressure
CRP: C Reactive Protein
CSF: Cerebro-Spinal Fluid
CT: Computed Tomography
EEG: Electroencephalogram
GAS: Group A Streptococcus
ICU: Intensive Care Unit
iGAS: invasive Group A Streptococcus infection
INR: International Normalized Ratio
MRI: Magnetic Resonance Imaging
NAAT: Nucleic Acid Amplification Techniques
Neu: Neutrophils
PT: Prothrombin Time
RADT: Rapid Antigen Detection Tests
SpO2: Saturation of Peripheral Oxygen
STSS: Streptococcal Toxic Shock Syndrome
VZV: Varicella Zoster Virus
WBC: White Blood Cells
WHO: World Health Organization

## References

[1] Kanwal S, Vaitla P, Streptococcus P. 2022 Aug 1. In: StatPearls [Internet]. Treasure Island (FL): StatPearls Publishing; 2022 Jan–. PMID: 32119415.

[2] Mustafa Z, Ghaffari M, Diagnostic Methods. Clinical guidelines, and Antibiotic Treatment for Group A Streptococcal Pharyngitis: a narrative review. Front Cell Infect Microbiol 2020 Oct 15;10:563627. doi: 10.3389/fcimb.2020.563627. PMID: 33178623; PMCID: PMC7593338. 10.3389/fcimb.2020.563627 PMC7593338 33178623

[3] Cunningham MW. Pathogenesis of group a streptococcal infections. ClinMicrobiol Rev. 2000 Jul;13(3):470–511. 10.1128/CMR.13.3.470. PMID: 10885988; PMCID: PMC88944. 10.1128/cmr.13.3.470-511.2000 PMC88944 10885988

[4] World Health Organization. (15 December 2022). Disease Outbreak News; Increased incidence of scarlet fever and invasive Group A Streptococcus infection—multi-country. Available at: https://www.who.int/emergencies/disease-outbreak-news/item/2022-DON429.

[5] SC MPC ATS Milano, Report Malattie Infettive ATS Milano Resoconto. 2022. Available at: https://www.ats-milano.it/sites/default/files/2023-02/Report%20malattie%20infettive%202022.pdf.

[6] Steer AC, Lamagni T, Curtis N, Carapetis JR. Invasive group a streptococcal disease: epidemiology, pathogenesis and management. Drugs. 2012 Jun 18;72(9):1213-27. doi: 10.2165/11634180-000000000-00000. PMID: 22686614; PMCID: PMC7100837. 10.2165/11634180-000000000-00000 PMC7100837 22686614

[7] Guy R, Henderson KL, Coelho J, Hughes H, Mason EL, Gerver SM, Demirjian A, Watson C, Sharp A, Brown CS, Lamagni T. Increase in invasive group a streptococcal infection notifications, England, 2022. Euro Surveill. 2023 Jan;28(1):2200942. doi: 10.2807/1560-7917.ES.2023.28.1.2200942. PMID: 36695450; PMCID: PMC9817207. 10.2807/1560-7917.ES.2023.28.1.2200942 PMC9817207 36695450

[8] De Gier B, de Beer-Schuurman I, de Melker H, van Sorge N. Increase in invasive Streptococcus pyogenes disease among young children and adults, the Netherlands, March-July 2022. European Scientific Conference on Applied Infectious Disease Epidemiology (ESCAIDE); 23–25 November 2022, Stockholm, Sweden. Poster presentation; Abstract ID: 511. Available from: https://www.escaide.eu/sites/default/files/documents/ESCAIDE2022_AbstractBook.pdf.

[9] SantéPublique F. Infection invasive à streptocoque du Groupe A (IISGA): point de situation au 6 décembre 2022. [Invasive group A streptococcal disease (IISGA): status update on 6 December 2022]. Saint-Maurice cedex: SantéPublique France; 2022. French. Available from: https://www.santepubliquefrance.fr/les-actualites/2022/infection-invasive-a-streptocoque-du-groupe-a-iisga-point-de-situation-au-6-decembre-2022.

[10] Health Protection Surveillance Centre (HPSC). Update on group A streptococcus. Dublin: HPSC. ; 2022. Available from: https://www.hpsc.ie/news/title-22663-en.html.

[11] European Centre for Disease Prevention and Control (ECDC). Communicable disease threats report, 4–10 December 2022, week 49. Stockholm: ECDC. ; 2022. Available from: https://www.ecdc.europa.eu/en/publications-data/communicable-disease-threats-report-410-december-2022-week-49.

[12] Centers for Disease Control and Prevention (CDC). Increase in invasive group A strep infections, 2022. Atlanta: CDC. ; 2022. Available from: https://www.cdc.gov/groupastrep/igas-infections-investigation.html#:~:text=CDC%20is%20looking%20into%20a,and%20streptococcal%20toxic%20shock%20syndrome.

[13] De Gier B, Marchal N, de Beer-Schuurman I, TeWierik M, Hooiveld M, ISIS-AR Study Group; GAS Study group, de Melker HE, van Sorge NM. Members of the GAS study group; members of the ISIS-AR study group. Increase in invasive group a streptococcal (Streptococcus pyogenes) infections (iGAS) in young children in the Netherlands, 2022. Euro Surveill. 2023 Jan;28(1):2200941. PMID: 36695447; PMCID: PMC9817208. 10.2807/1560-7917.ES.2023.28.1.2200941 PMC9817208 36695447

[14] Cohen JF, Bertille N, Cohen R, Chalumeau M. Rapid antigen detection test for group a streptococcus in children with pharyngitis. Cochrane Database Syst Rev 2016 Jul 4;7(7):CD010502. doi: 10.1002/14651858.CD010502.pub2. PMID: 27374000; PMCID: PMC6457926. 10.1002/14651858.CD010502.pub2 PMC6457926 27374000

[15] World Health Organization (12. December 2022). Increase in invasive Group A streptococcal infections among children in Europe, including fatalities. Available at: https://www.who.int/europe/news/item/12-12-2022-increase-in-invasive-group-a-streptococcal-infections-among-children-in-europe--including-fatalities.

[16] Cattoir V, Mechanisms of Streptococcus pyogenes Antibiotic Resistance. 2022 Sep 19 [updated 2022 Oct 9]. In: Ferretti JJ, Stevens DL, Fischetti VA, editors. Streptococcus pyogenes: Basic Biology to Clinical Manifestations [Internet]. 2nd ed. Oklahoma City (OK): University of Oklahoma Health Sciences Center; 2022 Oct 8. Chapter 30. PMID: 36479762. 36479762

[17] Meletis G, SoulopoulosKetikidis AL, Floropoulou N, Tychala A, Kagkalou G, Vasilaki O, Mantzana P, Skoura L, Protonotariou E. Antimicrobial resistance rates of Streptococcus pyogenes in a greek tertiary care hospital: 6-year data and literature review. New Microbiol. 2023 Feb;46(1):37–42. PMID: 36853816. 36853816

[18] Gajdács M, Ábrók M, Lázár A, Burián K, Beta-Haemolytic Group A. C and G streptococcal infections in Southern Hungary: a 10-Year Population-Based Retrospective Survey (2008–2017) and a review of the literature. Infect Drug Resist 2020 Dec 31;13:4739–49. doi: 10.2147/IDR.S279157. PMID: 33408489; PMCID: PMC7781025. 10.2147/IDR.S279157 PMC7781025 33408489

[19] Li H, Zhou L, Zhao Y (2020). Molecular epidemiology and antimicrobial resistance of group a streptococcus recovered from patients in Beijing, China. BMC Infect Dis.

