# Supplementary material for: Invasive Group A streptococcal infections: are we facing a new outbreak? A case series with the experience of a single tertiary center
Source: Ital J Pediatr. 2023 Jul 19;49:88. doi: 10.1186/s13052-023-01494-9 (PMC10354902; doi:10.1186/s13052-023-01494-9)
Supplement: Supplementary file 1 — Supplementary Material 1 [file 13052_2023_1494_MOESM1_ESM.pdf]

CARE case report guidelines

CARE Checklist of information to include when writing a case report

(cc) BY-NC-ND

Topic	Item	Checklist item description	Reported on Line
Title	1	The diagnosis or intervention of primary focus followed by the words "case report"	3
Key Words	2	2 to 5 key words that identify diagnoses or interventions in this case report, including "case report"	37
Abstract (no references)	3a	Introduction: What is unique about this case and what does it add to the scientific literature?	18-20
	3b	Main symptoms and/or important clinical findings	25
	3c	The main diagnoses, therapeutic interventions, and outcomes	25 - 29
	3d	Conclusion—What is the main "take-away" lesson(s) from this case?	32
Introduction	4	One or two paragraphs summarizing why this case is unique (**may include references**)	49 - 65
Patient Information	5a	De-identified patient specific information.	70 - 101 - 116 - 153
	5b	Primary concerns and symptoms of the patient.	71 - 102 - 117 - 154
	5c	Medical, family, and psycho-social history including relevant genetic information	70 - 101 - 116 - 153
	5d	Relevant past interventions with outcomes	70 - 101 - 116 - 153
Clinical Findings	6	Describe significant physical examination (PE) and important clinical findings.	71 - 102 - 117 - 154
Timeline	7	Historical and current information from this episode of care organized as a timeline	71 - 102 - 117 - 154
Diagnostic Assessment	8a	Diagnostic testing (such as PE, laboratory testing, imaging, surveys).	73 - 102 - 118 - 155
	8b	Diagnostic challenges (such as access to testing, financial, or cultural)	85 - 159
	8c	Diagnosis (including other diagnoses considered)	90 - 104 - 130 - 163
	8d	Prognosis (such as staging in oncology) where applicable	95 - 111 - 146 - 172
Therapeutic Intervention	9a	Types of therapeutic intervention (such as pharmacologic, surgical, preventive, self-care)	87 - 107 - 128 - 161
	9b	Administration of therapeutic intervention (such as dosage, strength, duration)	87 - 107 - 128 - 161
	9c	Changes in therapeutic intervention (with rationale)	87 - 107 - 128 - 161
Follow-up and Outcomes	10a	Clinician and patient-assessed outcomes (if available)	95 - 111 - 146 - 172
	10b	Important follow-up diagnostic and other test results	147 - 172
	10c	Intervention adherence and tolerability (How was this assessed?)	87 - 107 - 128 - 161
	10d	Adverse and unanticipated events	/
Discussion	11a	A scientific discussion of the strengths AND limitations associated with this case report	193 - 194 - 198 - 199
	11b	Discussion of the relevant medical literature **with references**.	182 - 184 - 187 - 193 - 200
	11c	The scientific rationale for any conclusions (including assessment of possible causes)	182 - 193 - 198
	11d	The primary "take-away" lessons of this case report (without references) in a one paragraph conclusion	208
Patient Perspective	12	The patient should share their perspective in one to two paragraphs on the treatment(s) they received	/
Informed Consent	13	Did the patient give informed consent? Please provide if requested	Yes ☒ No ☐
